# Reported Dietary Patterns in Pregnant Women with and Without Gestational Diabetes Mellitus: A Post-Diagnosis Comparative Study in Guadalajara, Mexico

**DOI:** 10.3390/healthcare14131819

**Published:** 2026-06-23

**Authors:** Andrea Paola Gómez-Maldonado, Laura Leticia Salazar-Preciado, Clío Chávez-Palencia, J. Jesús Pérez-Molina, Claudia Hunot-Alexander

**Affiliations:** 1 Instituto de Nutrición Humana, Centro Universitario de Ciencias de la Salud, Universidad de Guadalajara, Guadalajara 44340, Jalisco, Mexico; paola.gomez4084@alumnos.udg.mx (A.P.G.-M.); clio.chavez@academicos.udg.mx (C.C.-P.); 2 División de Ciencias de la Salud, Centro Universitario de Tonalá, Universidad de Guadalajara, Tonalá 45425, Jalisco, Mexico; 3 Departamento de Clínicas de la Reproducción Humana, Crecimiento y Desarrollo Infantil, Centro Universitario de Ciencias de la Salud, Universidad de Guadalajara, Guadalajara 44340, Jalisco, Mexico; j.pmolina@academicos.udg.mx

**Keywords:** dietary patterns, gestational diabetes mellitus, dietary assessment, health outcomes, principal component analysis, ultra processed foods, nutritional transition

## Abstract

**Background:** Gestational diabetes mellitus (GDM) affects between 1% and 14% of pregnancies worldwide. Major risk factors include advanced maternal age, excess adiposity, family history of type 2 diabetes, and unhealthy dietary habits. In Mexico, evidence on the association between dietary patterns and GDM remains scarce, particularly in socioeconomically vulnerable populations with limited access to specialized nutrition services. This study aimed to evaluate the association between dietary patterns and the presence of GDM in pregnant women attending the outpatient obstetrics clinic of a teaching public hospital in Guadalajara, México. **Methods:** We conducted a case–control study including 169 pregnant women: 71 with GDM confirmed by the ADA one-step 75 g oral glucose tolerance test OGTT criteria and 98 without GDM based on a negative OGTT, recruited consecutively from the same clinic during the same period. Dietary intake was assessed using a culturally adapted and validated Food Frequency Questionnaire. Dietary patterns were identified through Principal Component Analysis, and associations were examined using logistic regression adjusted for maternal age, pregestational BMI, and family history of type 2 diabetes. **Results:** Women with GDM had higher maternal age, greater pregestational BMI, and more frequent family history of type 2 diabetes compared with controls. Three dietary patterns were identified: Western, Healthy, and Dairy/Refined. High adherence to the Western pattern was inversely associated with GDM (aOR = 0.36; 95% CI: 0.16–0.78; *p* = 0.010); however, this finding most likely reflects post-diagnosis dietary modifications rather than a protective effect, while maternal age remained the strongest risk factor (OR = 1.09; 95% CI: 1.03–1.16; *p* = 0.002). The Healthy pattern (aOR = 1.25; 95% CI: 0.55–2.82; *p* = 0.593) and the Dairy/Refined pattern (aOR = 0.80; 95% CI: 0.39–1.66; *p* = 0.554) were not significantly associated with GDM in the adjusted model. **Conclusions:** GDM was associated with older maternal age, higher pregestational BMI, and family history of T2DM. The inverse association with the Western pattern may reflect post-diagnosis dietary changes rather than a protective effect. Due to the retrospective design, causal inference is not possible, highlighting the need for longitudinal studies.

## 1. Introduction

Gestational diabetes mellitus (GDM) is one of the most common metabolic complications of pregnancy and is associated with adverse maternal and neonatal outcomes, including preeclampsia, cesarean delivery, fetal macrosomia, and an increased long-term risk of type 2 diabetes mellitus for both mother and child [1,2,3,4,5]. The prevalence of GDM has increased worldwide in parallel with rising rates of overweight and obesity among women of reproductive age. In Mexico, obesity affects more than one-third of adult women and represents one of the main risk factors for GDM development [6,7,8,9,10,11,12,13]. Recent evidence from Mexico and Latin America also indicates that advanced maternal age, pregestational obesity, and family history of diabetes are among the most important determinants of GDM risk [7,8,9,10,11,12]. Given its growing prevalence and health consequences, GDM constitutes a significant public health challenge in the region.

Diet plays a central role in the prevention and management of GDM, and dietary interventions during pregnancy have been associated with improved maternal glycemic control and favorable infant birth weight outcomes compared with usual nutritional advice [14]. However, dietary behaviors are influenced not only by individual choices but also by broader social, economic, and environmental conditions. In Mexico, structural and socioeconomic determinants, including food insecurity, limited access to nutrition services, low educational attainment, and an obesogenic food environment, shape dietary behaviors during pregnancy and may amplify the impact of nutritional risk factors on GDM development [15,16,17,18,19,20,21,22,23,24,25]. These contextual factors are particularly relevant among women receiving care in public healthcare institutions, where individualized nutritional counseling may be limited, and dietary recommendations are often difficult to adapt to cultural practices and socioeconomic realities [16,26].

Mexico is currently undergoing a nutritional transition characterized by increased consumption of ultra-processed foods, sugar-sweetened beverages, and energy-dense products, alongside a declining intake of traditional foods such as legumes, fruits, vegetables, and whole grains [23,24,25,27,28]. These dietary changes have been associated with obesity, metabolic disorders, and poor glycemic outcomes, highlighting the importance of evaluating overall dietary patterns rather than isolated nutrients or foods [23,24,25,27,28,29]. Dietary pattern analysis provides a comprehensive approach to understanding habitual food consumption and its relationship with health outcomes in real-world settings.

Although previous studies have examined the association between dietary patterns and GDM in different populations, evidence from Mexico remains limited, particularly among socially vulnerable women receiving care in public health services [13,29,30]. Furthermore, little is known about how the ongoing nutritional transition and the structural conditions experienced by these populations may influence dietary patterns and their relationship with GDM risk. Therefore, this study aimed to identify and compare reported dietary patterns between pregnant women with and without GDM attending a public teaching hospital in Guadalajara, Jalisco, Mexico. To our knowledge, this is among the first studies to characterize dietary patterns in relation to GDM in a socially vulnerable, urban Mexican population attending a high-specialty public referral center. The findings may contribute to the development of culturally appropriate and feasible nutritional strategies for GDM management in resource-limited public healthcare settings in Mexico and Latin America.

## 2. Materials and Methods

### 2.1. Participants and Procedures

We conducted a retrospective analytical case–control study between March and December 2024 at the Maternal–Fetal Obstetrics outpatient clinic of a teaching public hospital located in Guadalajara, México. The case–control framework was used to compare self-reported dietary patterns between women with confirmed GDM (cases) and women without GDM (controls); given that dietary assessment was conducted after diagnosis, the associations identified in this study should be interpreted as descriptive comparisons of reported dietary behaviors rather than as evidence of etiological dietary risk factors for GDM.

The planned sample size (*n* = 146; 73 per group) was calculated a priori using Fleiss’ formula for unmatched case–control studies based on pilot estimates (controls exposed 60.8%, cases exposed 36.46%), α = 0.05, and 80% power [31]. Although the formula allows specifying different control-to-case ratios, these parameters indicated that a 1:1 allocation ratio was sufficient to achieve the desired statistical power. Several case–control studies evaluating dietary patterns using PCA have adopted similar 1:1 ratios justified through Fleiss-based calculations [32,33], supporting the methodological appropriateness of our design.

Recruitment followed non-probabilistic consecutive sampling, including all eligible women who attended the Maternal–Fetal Obstetrics clinic. The clinic social worker provided access to the clinical records of women attending the service, while eligibility was assessed by the principal investigator according to the predefined inclusion and exclusion criteria. Women meeting the eligibility criteria were subsequently invited to participate. Of these, 169 women met all inclusion criteria, agreed to participate, and completed the study procedures, resulting in a final analytical sample of 71 cases and 98 controls. The primary reasons for non-inclusion were failure to meet one or more inclusion criteria (e.g., pre-existing metabolic conditions, multiple pregnancy, or substance use) and refusal to participate. No participant withdrew after beginning the interview. A flow diagram summarizing the screening, eligibility assessment, and final sample selection is provided in Figure S1. Once eligibility was confirmed, the principal investigator informed the women about the study objectives and invited those interested to participate; written informed consent was obtained from all who agreed. The cases were pregnant women with a confirmed diagnosis of GDM based on the ADA one-step 75 g OGTT criteria [34], while the controls were pregnant women attending the same clinic during the same period with a negative OGTT. Both groups met the same inclusion and exclusion criteria and were recruited consecutively. Inclusion criteria were women aged >18 years, with singleton pregnancies, and under prenatal follow-up. Exclusion criteria included pre-existing metabolic diseases, chronic conditions requiring continuous medical care, or alcohol, tobacco, or drug use that could interfere with glucose metabolism. The main difference between groups was that women classified as cases had a confirmed diagnosis of GDM and received standardized nutritional information in the form of a leaflet with generalized lists of foods to eat and avoid, a non-individualized approach, whereas those in the control group had a negative diagnosis for GDM and only received general prenatal care recommendations.

Diagnosis of GDM was established through an oral glucose tolerance test (OGTT) according to the American Diabetes Association (ADA) criteria [34] between 24 and 28 weeks of gestation using the one-step 75 g OGTT, with glucose measured at fasting, 1 h, and 2 h. GDM was diagnosed when ≥1 of the following thresholds was met or exceeded: fasting ≥ 92 mg/dL; 1 h ≥ 180 mg/dL; 2 h ≥ 153 mg/dL [34].

The FFQ was administered after GDM diagnosis in both groups, during a subsequent prenatal visit at the Maternal–Fetal Obstetrics clinic. At the time of the interview, women with GDM had already received routine dietary recommendations as part of their clinical follow-up. Because dietary intake was assessed after diagnosis, it was not possible to determine whether the reported dietary patterns reflected pre-diagnosis eating habits or dietary modifications adopted following medical or nutritional counseling. This limitation should be considered when interpreting the observed associations, particularly the inverse association observed for the Western dietary pattern. This hospital is a high-specialty referral center that receives women only after an abnormal OGTT result from primary or secondary care; the FFQ was therefore administered at the earliest feasible point after referral. This referral-based dynamic explains why most participants were already in the third trimester at the time of data collection: 91.5% of cases and 88.8% of controls were ≥28 weeks of gestation when the FFQ and anthropometric assessments were conducted.

The study protocol was approved by the Ethics Committee of the Hospital Civil de Guadalajara (approval number 17C114-05/03/2024) and was classified as minimal risk according to the Mexican General Health Law on Health Research [35]. All participants were enrolled voluntarily, and procedures were conducted in accordance with the principles of the Declaration of Helsinki and the Guidelines of the Council for International Organizations of Medical Sciences (CIOMS) [36,37].

### 2.2. Variables and Data Collection

Data collection was conducted by the principal investigator (A.P.G.-M.) using a structured questionnaire. Socioeconomic status was assessed using the index developed by the Mexican Association of Market Intelligence and Opinion Agencies (AMAI), a tool widely used in Mexico for the socioeconomic classification of households [38]. The resulting categories were grouped for analytical purposes into high (A/B, C+) and low (C, C−, D+, D, and E). Educational attainment was recorded through interviews and subsequently grouped into groups for statistical analysis. Occupation was recorded and classified according to the National Institute of Statistics and Geography (INEGI) of Mexico [39], grouped for analytical purposes. Marital status was recorded and subsequently categorized according to cohabitation with a partner for the corresponding analyses. Place of residence was classified as the Guadalajara Metropolitan Area or other localities.

Anthropometric variables included current weight, pregestational weight (retrieved from medical records), pregestational body mass index (BMI), and gestational weight gain, considered as a continuous variable. Gyneco-obstetric variables included family history of T2DM (yes/no); gestational age at the time of the survey, categorized as second trimester (14–27 weeks) or third trimester (≥28 weeks); and number of previous pregnancies, categorized as 0–2 or ≥3.

Current anthropometric measurements, including weight and height, were obtained following the International Society for the Advancement of Kinanthropometry (ISAK) protocol [40]. Weight was measured using a TANITA electronic scale (model BF-679F), and height was assessed with a portable SECA stadiometer (model 213). Participants were evaluated barefoot, wearing light clothing, in the anatomical position, with the head aligned in the Frankfurt plane. All measurements were performed by the principal investigator (A.P.G.-M.), a qualified nutritionist. BMI was calculated as weight in kilograms divided by height in meters squared (kg/m^2^) and categorized as underweight/normal weight (18.5–24.9 kg/m^2^), overweight (25.0–29.9 kg/m^2^), or obesity (≥30.0 kg/m^2^) [41].

### 2.3. Dietary Patterns

Dietary patterns were assessed using a qualitative, weekly Food Frequency Questionnaire (FFQ) specifically developed for the study population. The FFQ was constructed based on a detailed analysis of 24 h dietary recalls collected by a trained nutritionist between January and June 2022 from pregnant women attending the same Maternal–Fetal Obstetrics service from week 24 of gestation onward, as part of a master’s thesis conducted in the same population [42]. Food items were grouped according to their nutritional characteristics and adapted to the cultural and physiological context of pregnant women in Mexico. The preliminary version of the instrument was reviewed by the master’s thesis advisory committee, composed of nutrition specialists, to evaluate the relevance, clarity, and cultural appropriateness of the food items included. To ensure content clarity and cultural appropriateness, the instrument underwent cognitive interviewing (think-aloud technique) with 40 pregnant women prior to finalization, following current methodological recommendations for questionnaire development [43]. A pilot phase was conducted before data collection to evaluate administration procedures, and reproducibility was assessed through a test–retest procedure. Formal criterion validity against a reference method (e.g., repeated 24 h dietary recalls) was not performed; this constitutes a methodological limitation and is explicitly acknowledged. The instrument is therefore described as culturally developed and content-evaluated, consistent with the procedures undertaken.

The FFQ included 12 general food groups: (1) cereals and tubers, (2) vegetables, (3) fruits, (4) legumes, (5) meat and eggs, (6) fish and seafood, (7) dairy products, (8) fats, (9) traditional Mexican dishes (antojitos), (10) snack foods, sweets, and fast food, (11) beverages, and (12) sauces and condiments. Frequency response options ranged from never to ≥6 times per day. The FFQ was administered in a standardized manner through face-to-face interviews conducted by the principal investigator (A.P.G.-M.). Participants reported their frequency of consumption during the previous seven days.

Because women diagnosed with GDM were referred to the Maternal–Fetal Obstetrics clinic for specialized high-risk pregnancy follow-up, the FFQ was administered during a subsequent prenatal visit rather than at the time of diagnosis. Consequently, dietary data were collected after GDM diagnosis, and it was not possible to ascertain whether the reported dietary patterns reflected pre-diagnosis eating habits or dietary modifications adopted following medical or nutritional counseling during prenatal care. This limitation should be considered when interpreting the observed associations.

Food items with low consumption variability were excluded from the analysis because they provided limited discrimination among participants and contributed minimally to the identification of dietary patterns using PCA (coffee and tea, plain water, offal, vegetable oils, and non-processed sauces). Subsequently, items that did not load onto any component were also excluded (seafood, mature cheeses, legumes, tubers, eggs, and homemade bread). This sequential exclusion process, combining a priori exclusion of low variability items with a posteriori exclusion of items with insufficient loadings, is consistent with standard practice in PCA-based dietary research. After these exclusions, 24 food subgroups were analyzed through PCA with Varimax rotation (Table S1, Supplementary Materials). The FFQ demonstrated adequate discriminatory capacity for PCA (KMO = 0.723; Bartlett’s test *p* < 0.001).

### 2.4. Statistical Analysis

Normality was assessed using the Kolmogorov–Smirnov test. Quantitative variables were expressed as mean ± standard deviation (SD) or median and interquartile range (IQR), depending on data distribution. Categorical variables were expressed as absolute frequencies and percentages.

Between-group differences were analyzed using Student’s *t* test or the Mann–Whitney U test, according to data distribution. Associations between categorical variables were evaluated with the chi-square test, and odds ratios (OR) with 95% confidence intervals (CI) were additionally calculated. Correlations between quantitative variables were assessed using Pearson’s or Spearman’s correlation tests, depending on normality.

For bivariate analyses, maternal age was additionally categorized using the sample median (≥27 vs. <27 years) to facilitate interpretation of crude odds ratios. In multivariable logistic regression models, maternal age was entered as a continuous variable (per one-year increase) to maximize statistical efficiency and preserve information. Pregestational BMI was analyzed as a categorical variable (normal weight/underweight, overweight, and obesity), using normal weight/underweight as the reference category.

Dietary patterns were identified through Principal Component Analysis (PCA) with Varimax rotation, based on weekly food frequency data converted into numeric values. Model adequacy was assessed using the Kaiser-Meyer-Olkin (KMO) index and Bartlett’s test of sphericity. Food items with homogeneous consumption across participants, including coffee and tea, plain water, offal, and vegetable oils, were excluded prior to the analysis, given their lack of discriminatory variability. Items that did not load onto any component at or above the established threshold (factor loading ≥ 0.30) were excluded following the initial analysis; these included seafood, mature cheeses, legumes, tubers, eggs, and homemade bread. Factors with eigenvalues ≥1.5 were retained, applying a more conservative threshold than the classical Kaiser criterion (≥1.0), to minimize the inclusion of weak or poorly interpretable components. This criterion was applied in conjunction with examination of the scree plot and assessment of the substantive interpretability of each factor. Factor loadings below 0.30 were suppressed to facilitate identification of the food groups with substantive association to each pattern. Factor scores were categorized into quartiles; Q1 and Q2 were grouped as low adherence, and Q3 and Q4 as high adherence. This grouping strategy was adopted to improve model stability given the available sample size. Multivariable binary logistic regression models were constructed to evaluate the association between dietary patterns and the presence of GDM. Variables were entered using the forward conditional method, and models were adjusted for risk factors identified in the bivariate analysis (*p* < 0.05), including maternal age, pregestational BMI, and family history of T2DM. Other sociodemographic and obstetric variables were considered during the analytical process but were not included in the final model because they did not substantially modify the association estimates and maintain a model appropriate for the available sample size. Model fit was evaluated using the Hosmer–Lemeshow goodness-of-fit test. Results were expressed as adjusted odds ratios (aOR) with 95% CI.

All statistical analyses were performed using SPSS software, version 28 (IBM Corp., Armonk, NY, USA). A *p*-value < 0.05 was considered statistically significant.

## 3. Results

### 3.1. General Description and Comparative Analysis of the Samples

Although the estimated sample size was 74 participants per group, only 71 cases (42%) met all inclusion criteria during the established fieldwork period. In the control group, 98 eligible women (58%) were recruited, resulting in a total of 169 pregnant women. This variation did not compromise group comparability and slightly increased the statistical power of the analysis.

The sociodemographic and anthropometric characteristics of the participants are presented in Table 1. Women with GDM were older and had higher pregestational BMI than those without GDM. In addition, a family history of T2DM was more common among women with GDM. Detailed sociodemographic and anthropometric characteristics are presented in Table 1.

Nearly half of women with GDM (47.9%) were living with pregestational obesity, compared with 24.5% in the control group (*p* < 0.001). When obesity was compared with non-obesity (normal weight + overweight), women living with pregestational obesity showed nearly a threefold increase in the odds of developing GDM (OR = 2.83; 95% CI: 1.47–5.44; *p* = 0.002). In contrast, no significant differences were observed between overweight and obesity categories (OR = 0.68; 95% CI: 0.32–1.43; *p* = 0.313).

A moderate negative correlation was observed between pregestational BMI and gestational weight gain (rho = −0.356; *p* < 0.001). No significant differences were found between groups in height or gestational weight gain (*p* > 0.05).

Regarding categorical sociodemographic variables, both groups showed similar distributions. Most participants reported low socioeconomic status (77%), being homemakers (72%), and residing in the metropolitan area of Guadalajara (>70%). Although a higher proportion of women with GDM lived with a partner (88.7% vs. 77.6%) and had slightly higher educational attainment compared with controls (15.5% vs. 11.2%), these differences were not statistically significant (*p* > 0.05).

A family history of T2DM was significantly more frequent in the GDM group (81.7% vs. 58.2%; *p* < 0.001), conferring a 3.21-fold increased probability of developing the condition (95% CI: 1.56–6.61). No significant associations were observed with the number of previous pregnancies (*p* = 0.226). The median gestational age at OGTT was 33 weeks (IQR: 30–35), with no statistically significant difference between groups (*p* = 0.554).

To enhance the interpretability of gestational weight gain (GWG), GWG per week (kg/week) was additionally calculated as a descriptive metric. The mean (±SD) GWG per week was 0.270 ± 0.205 kg/week in the non-GDM group and 0.226 ± 0.200 kg/week in the GDM group, with corresponding medians of 0.265 (IQR: 0.154–0.376) and 0.222 (IQR: 0.096–0.348), respectively.

### 3.2. Identification of Dietary Patterns

The original FFQ included 35 items distributed across 12 general food groups. For the analysis, items with low variability or homogeneous consumption among participants (coffee and tea, plain water, offal, vegetable oils, and non-processed sauces) were excluded, as well as those that did not load onto any component, such as seafood, mature cheeses, legumes, tubers, eggs, and homemade bread. After these exclusions, 24 food subgroups remained and were analyzed through PCA with Varimax rotation. Sampling adequacy was acceptable (KMO = 0.723; Bartlett’s test of sphericity *p* < 0.001). Three dietary patterns were identified: the Western pattern explained 17.5% of the variance, the Healthy pattern explained 11.1%, and the Dairy/Refined pattern explained 7.0%, accounting for a total explained variance of 35.5%. The scree plot revealed a sharp decline after the third component, supporting the retention of three factors (Figure S2, Supplementary Materials).

The first pattern, referred to as the Western pattern, was characterized by high consumption of snack foods, sugar-sweetened beverages, traditional Mexican dishes (*antojitos*), processed sauces, processed meats, and fast food. The second, identified as the Healthy pattern, was composed mainly of fruits, vegetables, lean protein sources, and whole grains. The third, labeled the Dairy/Refined pattern, was defined by foods such as milk, yogurt, bread, and refined cereals.

Table 2 presents the rotated factor loading matrix, which details the food groups contributing to each dietary pattern and the magnitude of their association with the identified factors. To facilitate interpretation, a column reporting the percentage of variance explained by each factor could be included.

### 3.3. Adherence to Dietary Patterns

When adherence was analyzed by quartile distribution, a higher proportion of cases was concentrated in the first quartile (Q1) for both the Western and Dairy/Refined patterns, indicating low adherence. In contrast, adherence to the Healthy pattern was more evenly distributed across quartiles, with a tendency toward concentration in the second and third quartiles (Q2 and Q3) (Table 3).

Table 4 presents the qualitative comparison of quartiles according to adherence level to the three dietary patterns between cases and controls. Statistically significant differences were observed in adherence to the Western (*p* = 0.002) and Dairy/Refined (*p* = 0.032) patterns, with lower adherence among women with GDM. Conversely, adherence to the Healthy pattern was higher in cases, although no significant differences were found between groups (*p* = 0.825).

### 3.4. Bivariate Analysis of the Association Between Dietary Patterns and Gestational Diabetes Mellitus

The chi-square analysis showed that all three identified dietary patterns were significantly associated with the presence of GDM: the Western pattern (*p* < 0.001), followed by the Dairy/Refined pattern (*p* = 0.003) and the Healthy pattern (*p* = 0.014). A significant linear trend was also observed for the Western and Dairy/Refined patterns, whereas the Healthy pattern did not display a consistent trend (Table 5).

In addition, the analysis of dietary pattern factor scores between women with and without GDM revealed statistically significant differences, particularly for the Western (*p* = 0.002) and Dairy/Refined (*p* = 0.001) patterns, confirming previous findings.

### 3.5. Multivariable Analysis of the Association Between Dietary Patterns and Gestational Diabetes Mellitus

A binary logistic regression analysis adjusted for maternal age, pregestational BMI, and family history of T2DM was performed to evaluate the association between dietary patterns and the presence of GDM.

Table 6 presents the multivariable model constructed using binary logistic regression, with GDM as the dependent variable. The model was adjusted for maternal age, pregestational BMI, and family history of T2DM, based on their clinical relevance and their association with GDM in the bivariate analyses (*p* < 0.05). The model demonstrated good fit according to the Hosmer–Lemeshow test (χ^2^ = 4.494; *p* = 0.810).

In the crude analysis, the Western dietary pattern was inversely associated with GDM (cOR = 0.38; 95% CI: 0.20–0.72), and this association remained significant after adjustment (aOR = 0.36; 95% CI: 0.16–0.78; *p* = 0.010). In contrast, the Dairy/Refined pattern showed a crude association with GDM (cOR = 0.51; 95% CI: 0.27–0.95), but this association was no longer significant in the adjusted model (aOR = 0.80; 95% CI: 0.39–1.66; *p* = 0.554). The Healthy pattern was not significantly associated with GDM in either the crude or adjusted analyses.

Because dietary information was collected after the diagnosis of GDM, this association may have been influenced by dietary modifications resulting from nutritional treatment.

## 4. Discussion

This study analyzed dietary patterns and their association with GDM in a sample of 169 pregnant women attending a public Maternal–Fetal Obstetrics service in Guadalajara. Three patterns were identified, Western, Healthy, and Dairy/Refined, reflecting the coexistence of traditional and increasingly Westernized eating behaviors in Mexico. The relationships between these patterns and GDM provide relevant evidence on modifiable risk factors in a population characterized by structural vulnerability.

The findings of this study confirm that women with GDM were older and had higher pregestational BMI, both of which are well-established predictors of impaired glucose metabolism [6,7,8,9,10,11,12]. In addition, the high frequency of low socioeconomic status, homemaker occupation, and residence in the metropolitan area of Guadalajara reflects a context of structural vulnerability [44]. Within this context, the dietary patterns identified in this population may reflect the ongoing nutritional transition in Mexico, characterized by the coexistence of traditional dietary practices and increased consumption of ultra-processed foods, sugar-sweetened beverages, and refined products, alongside a decline in traditional foods such as legumes, fruits, and vegetables [23,24,25,27,28,45].

The inverse association observed between the Western dietary pattern and GDM should not be interpreted as a protective effect. Because dietary information was collected after the diagnosis of GDM, the observed inverse association with the Western dietary pattern may reflect dietary modifications following the initiation of prenatal follow-up and nutritional treatment rather than a true protective effect. Since post-diagnosis dietary changes were not directly measured, this interpretation remains speculative, and the finding should be considered an observational association rather than evidence of causality. Women diagnosed with GDM in this clinical setting frequently adopt restrictive eating behaviors influenced by generalized dietary recommendations and food restriction lists, which may reduce the reported consumption of snacks, sugar-sweetened beverages, processed foods, and other foods perceived as harmful. Therefore, this finding should be interpreted cautiously and not as evidence that adherence to a Western dietary pattern reduces GDM risk.

The Healthy pattern did not show a significant association with GDM in the adjusted model. Although its components are commonly linked to metabolic benefits, protective associations tend to emerge in longitudinal designs or when dietary exposures are assessed prior to pregnancy [45,46,47]. The Dairy/Refined pattern showed a positive association in crude analyses that was attenuated after adjustment. This finding may reflect the opposing metabolic properties of its components, including dairy products, which have been associated with favorable metabolic outcomes in some studies [48], and refined cereals, which have consistently been associated with higher glycemic load and increased GDM risk [49]. Higher fiber intake and traditional dietary components, including legumes, whole grains, and vegetables, have also been associated with better glycemic control in women with GDM [45,50], underscoring the importance of examining real-world dietary behaviors in this population.

Within the healthcare setting where this study was conducted, access to specialized nutritional care may be limited, and many women may receive general dietary recommendations, which could hinder adherence to individualized nutritional therapy [47,51,52]. This context highlights the importance of providing culturally appropriate nutritional counseling early in prenatal care to support sustainable dietary changes and avoid unnecessarily restrictive recommendations [47,53]. Overall, these findings contribute to the growing evidence base supporting the development of context-specific nutritional strategies for women at risk of GDM in Mexico and other Latin American settings [47,51,52,53].

One limitation of this study is that the FFQ was administered after the diagnosis of GDM, which may have influenced participants’ reporting of dietary intake. In this context, recall bias and the underreporting of foods perceived as unhealthy, such as snacks, sugar-sweetened beverages, or traditional dishes, are possible among women with GDM and may partly explain the inverse association observed for the Western dietary pattern.

Although the FFQ was culturally adapted and pilot tested using cognitive interviewing, it was not formally validated against repeated 24 h dietary recalls. Therefore, measurement error cannot be ruled out. In addition, the FFQ captured weekly consumption frequency without portion size estimation or total energy intake assessment. This precludes adjustment for total caloric intake in the regression models, a recognized limitation in nutritional epidemiology. While PCA-based dietary pattern identification can be conducted validly using frequency data [46], the absence of energy and portion data limits the ability to adjust for dietary density, to estimate absolute nutrient intakes, and to make quantitative comparisons with studies employing weighed food records or quantitative 24 h dietary recalls.

Several potentially relevant confounding variables, including physical activity, previous nutritional counseling outside the study setting, and pharmacological or insulin treatment, were not measured and therefore could not be incorporated into the adjusted models. Residual confounding cannot be excluded.

Furthermore, the retrospective nature inherent to the case–control design limits the ability to infer temporal or causal relationships. Dietary assessment based on a single 7-day retrospective FFQ may not fully capture changes in dietary behaviors throughout pregnancy.

Finally, the use of non-probabilistic consecutive sampling in a single referral healthcare center limits the generalizability of the findings to other obstetric populations. Because systematic comparative information between included and non-included women was not available, a formal assessment of potential selection bias could not be performed. Women reporting current alcohol, tobacco, or drug use were excluded due to their potential effects on glucose metabolism and GDM risk, which may further limit generalizability.

## 5. Conclusions

This study described and compared self-reported dietary patterns between pregnant women with and without GDM attending a public teaching hospital in Guadalajara, Mexico. Three dietary patterns were identified: Western, Healthy, and Dairy/Refined, reflecting the coexistence of traditional and Westernized dietary behaviors in this socially vulnerable population. Maternal age, pregestational BMI, and family history of T2DM were more prevalent among women with GDM, consistent with established risk factors. An inverse association between the Western pattern and GDM was observed in the adjusted model; however, this finding is best attributed to post-diagnosis dietary modification rather than to a pre-existing etiological exposure, given the post-diagnosis timing of dietary assessment. No significant associations were observed for the Healthy or Dairy/Refined patterns in the adjusted analysis. Given these methodological constraints, no causal inference can be drawn from these findings. Longitudinal studies assessing dietary patterns from early gestation or the preconception period are needed to establish the role of diet in GDM risk in this population and to inform the development of culturally relevant, individually tailored nutritional interventions.

## Figures and Tables

**Table 1:** Comparison of sociodemographic and anthropometric quantitative variables between cases (*n* = 71) and controls (*n* = 98), according to data distribution.

Variables	Cases (*n* = 71)		Controls (*n* = 98)		*p*
	Median	(IQR)	Median	(IQR)	
Sociodemographic					
Age (years)	30 *	6	25	(21–31)	<0.001 †
AMAI score	132 *	40.7 *	135 *	39.4 *	0.628
Anthropometric					
Pregestational weight (kg)	75	(65–96)	63.5	(56–79)	<0.001 †
Current weight (kg)	87.4 *	16.5 *	72 *	(64.5–87.6)	<0.001 †
Pregestational BMI (kg/m^2^)	29.9	(26.6–36.7)	25.1	(21.9–29.7)	<0.001 †
Height (cm)	159.3 *	5.6 *	159 *	6.3 *	0.898
Gestational weight gain (kg)	7.4 *	6.3 *	8.7 *	6.5 *	0.210

AMAI: Mexican Association of Market and Opinion Research Agencies (range: 0–204 points; higher scores indicate higher socioeconomic level); cm: centimeters; kg: kilograms; kg/m^2^: kilograms per square meter; IQR: interquartile range; * Mean ± SD: mean and standard deviation; Statistical test: Student’s *t* test; † Mann–Whitney U test. Weight and GWG reflect measurements at the FFQ visit; GWG comparisons were also adjusted for gestational age at assessment.

**Table 2:** Rotated component loading matrix of food groups from the Food Frequency Questionnaire.

Dietary Pattern	Food Groups	Components			Explained Variance (%)
		1	2	3	
Western pattern					
17.5%	Savory snacks	0.675			17.5%
	Soft drinks	0.585			
	Processed sauces	0.581			
	Processed meats	0.570			
	Traditional Mexican dishes (*antojitos*)	0.563			
	Fast food	0.546			
	Sweets and candies	0.506			
	Red meat	0.460			
	Animal fats	0.431			
	Sugar-sweetened non-dairy beverages	0.373		0.352	
Healthy pattern					
	Nuts and seeds		0.701		11.1%
	Whole grains		0.630		
	Fish		0.562		
	Natural juices		0.556	0.335	
	Vegetables		0.511	−0.409	
	Fruits		0.506		
	Poultry		0.417		
	Fresh cheeses		0.308		
Dairy/Refined pattern					
	Whole dairy products			0.728	7.0%
	Bread	0.389		0.591	
	Skim dairy products			−0.587	
	Sugar-sweetened dairy beverages			0.538	
	Refined cereals			0.469	
	Tortilla			0.310	
Total					35.5%

**Table 3:** Distribution of adherence to dietary patterns by quartiles (Q1–Q4) in women with and without gestational diabetes mellitus.

Dietary Pattern	Quartile	Cases	Controls	Total
		*n*	*n*	
Western	Q1	31	11	42
	Q2	15	28	43
	Q3	8	34	42
	Q4	17	25	42
	Total	71	98	169
Healthy	Q1	13	29	42
	Q2	22	21	43
	Q3	24	18	42
	Q4	12	30	42
	Total	71	98	169
Dairy/Refined	Q1	28	14	42
	Q2	15	29	44
	Q3	14	28	42
	Q4	14	27	41
	Total	71	98	169

**Table 4:** Frequency and percentage of adherence to each dietary pattern and chi-square test.

Dietary Patterns	Cases (*n* = 71)		Controls (*n* = 98)		*p*
	*n*	%	*n*	(%)	
Western pattern					0.002
Low adherence	45	63.4	39	39.8	
High adherence	26	36.6	59	60.2	
Healthy pattern					0.825
Low adherence	35	49.3	50	51	
High adherence	36	50.7	48	49	
Dairy/Refined pattern					0.032
Low adherence	43	60.6	43	43.9	
High adherence	28	39.4	55	56.1	

**Table 5:** Association between dietary patterns and the presence of gestational diabetes mellitus, chi-square test.

Dietary Patterns	χ^2^ (df)	*p*	Linear Trend (*p*)	Contingency Coefficient
Western	27.46 (3)	<0.001	0.001	0.374
Healthy	10.65 (3)	0.014	0.968	0.243
Dairy/Refined	13.95 (3)	0.003	0.004	0.276

df: degrees of freedom.

**Table 6:** Association between dietary patterns and the presence of gestational diabetes mellitus in pregnant women *.

Variables	cOR (CI 95%)	*p*	aOR (CI 95%)	*p*
Dietary patterns				
Western pattern (Q4 vs. Q1)	0.38 (0.20–0.72)	0.002	0.36 (0.16–0.78)	0.010
Healthy pattern (Q4 vs. Q1)	1.07 (0.58–1.97)	0.825	1.25 (0.55–2.82)	0.593
Dairy/Refined pattern (Q4 vs. Q1)	0.51 (0.2–−0.95)	0.032	0.80 (0.39–1.66)	0.554
Covariates				
Age (years)	3.45(1.80–6.61)	<0.001	1.09 (1.03–1.16)	0.002
Pregestational BMI (Overweight vs. Normal weight)	1.93 (1.26–3.61)	0.004	1.73 (0.69–4.34)	0.239
Pregestational BMI (Obesity vs. Normal weight)	6.45 (2.65–15.63)	<0.001	2.23 (0.85–5.84)	0.101
Family history of T2DM (Yes vs. No)	3.21 (1.56–6.61)	<0.001	2.01 (0.86–4.69)	0.107

* Binary logistic regression adjusted for maternal age (continuous), pregestational BMI (categorical), and family history of T2DM. aOR: adjusted odds ratio; CI: confidence interval; cOR: crude odds ratio obtained from bivariate analysis; Q1: quartile 1; Q4: quartile 4. Note: In the crude model, age is presented as a dichotomous variable (≥27 vs. <27 years, based on the sample median) to facilitate bivariate comparisons consistent with published case–control studies on GDM; in the adjusted model, age was entered as a continuous variable (per one-year increase) to maximize statistical efficiency. Pregestational BMI is categorized as normal weight/underweight, overweight, and obesity in both models, using normal weight/underweight as the reference category. Hosmer-Lemeshow goodness-of-fit test: chi-square = 4.494, df = 8, *p* = 0.810.

## Data Availability

Data may be made available from the corresponding author upon reasonable request, subject to ethical and confidentiality considerations.

## Supplementary Materials

The following supporting information can be downloaded at: https://www.mdpi.com/article/10.3390/healthcare14131819/s1, Table S1. Food subgroups used in the Principal Component Analysis based on the food frequency questionnaire of 169 pregnant women, Figure S1. Participant recruitment, eligibility assessment and analytical sample flowchart, Figure S2. Principal component analysis (PCA) flowchart—identification of dietary patterns (DP’s), Figure S3. Scree plot of the Principal Component Analysis.

## Abbreviations

The following abbreviations are used in this manuscript:

ADA	American Diabetes Association
AMAI	Mexican Association of Market and Public Opinion Research Agencies
BMI	Body Mass Index
ENSANUT	National Health and Nutrition Survey (Encuesta Nacional de Salud y Nutrición)
FFQ	Food Frequency Questionnaire
GDM	Gestational Diabetes Mellitus
OGTT	Oral Glucose Tolerance Test
OR	Odds Ratio
PCA	Principal Component Analysis
T2DM	Type 2 Diabetes Mellitus
