# Supplementary material for: Reported Dietary Patterns in Pregnant Women with and Without Gestational Diabetes Mellitus: A Post-Diagnosis Comparative Study in Guadalajara, Mexico
Source: Healthcare (Basel). 2026 Jun 23;14(13):1819. doi: 10.3390/healthcare14131819 (PMC13360648; doi:10.3390/healthcare14131819)

Reported Dietary Patterns in Pregnant Women with and Without Gestational Diabetes Mellitus: A Post-Diagnosis Comparative Study in Guadalajara, Mexico

Andrea Paola Gómez-Maldonado, Laura Leticia Salazar-Preciado, Clío Chávez-Palencia, J. Jesús Pérez-Molina, Claudia Hunot-Alexander

Table S1. Food subgroups used in the Principal Component Analysis based on the food frequency questionnaire of 169 pregnant women.

Number	Food Group	Examples
1	Savory snacks	Chips, peanuts, pork rinds
2	Soft drinks	Soda
3	Processed sauces	Valentina, tajín, ketchup, chamoy, tomato purée
4	Processed meats	Sausage, ham, chorizo
5	Mexican traditional dishes	Sopes, flautas, torta ahogada, fried tacos, gorditas
6	Fast food	Pizza, hot dogs, hamburgers, breaded foods
7	Sweets and candies	Chocolate, Nutella, lollipops, candies, spicy candies, quince paste, flan
8	Red meats	Beef, pork
9	Animal fats	Cream, mayonnaise, butter, lard
10	Sugar-sweetened non-dairy beverages	Flavored waters (aguas frescas), packaged juices, Ades, coffee, tea
11	Nuts and seeds	Peanuts, pumpkin seeds, almonds, walnuts, sesame, natural peanut butter
12	Whole grains	Whole-grain bread, rice or pasta, rolled oats, plain popcorn, corn on the cob
13	Fish	Fresh fish, tuna
14	Natural juices	Fruit and vegetable juices
15	Vegetables	Carrot, zucchini, onion, tomato, peas, cactus (nopal), lettuce, cabbage, broccoli, cauliflower, mushroom, beet, green beans, chili peppers, bell peppers

16	Fruits	Banana, papaya, apple, orange, grapes, tangerine, mango, guava, watermelon, cantaloupe
17	Poultry	Chicken
18	Fresh cheeses	Panela, adobera, table cheese
19	Whole dairy products	Milk, yogurt
20	Bread	Sweet bread from local bakeries, homemade cookies, homemade cake
21	Skim dairy products	Skim milk, low-fat yogurt
22	Sugar-sweetened dairy beverages	Chocomilk, frappuccinos, mocha, atole
23	Refined grains	White bread, white rice or pasta, boxed cereal, birote (similar to baguette), baguette, flour, pancakes, flour tortillas
24	Tortilla	Corn tortilla

Figure S1. Participant recruitment, eligibility assessment and analytical sample flowchart

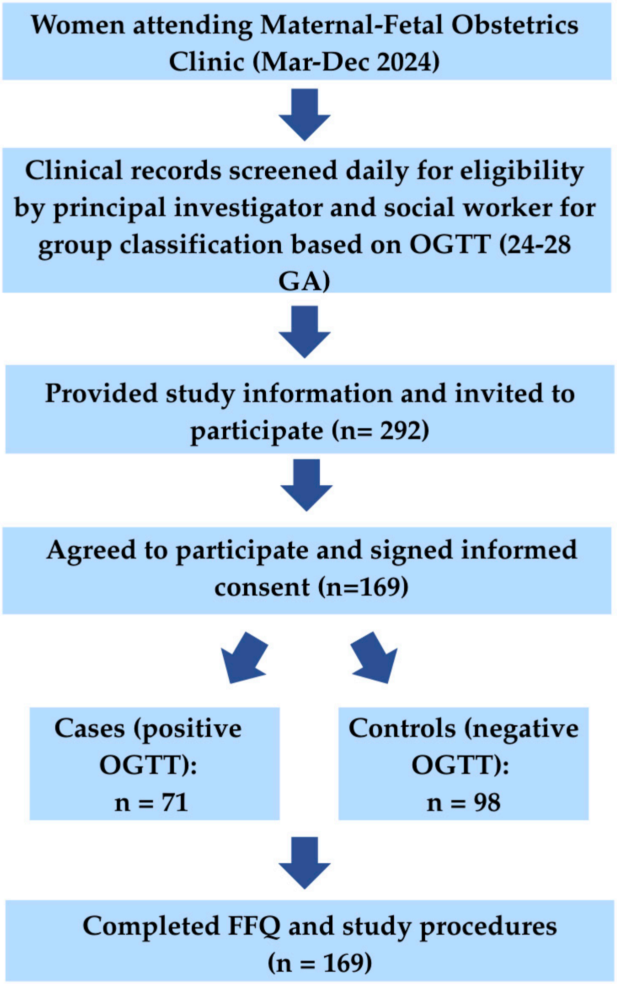

Figure S2. Principal component analysis (PCA) flowchart — identification of dietary patterns (DP´s)

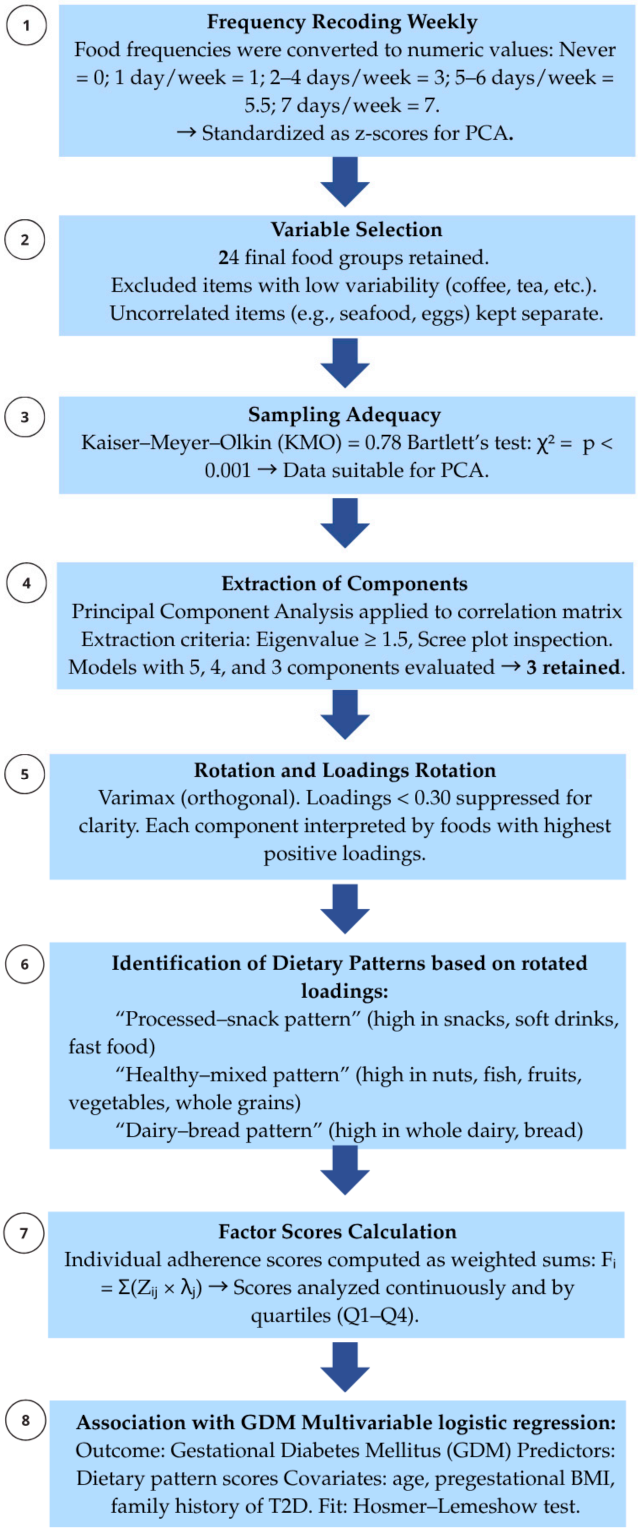

Figure S3. Scree plot of the Principal Component Analysis

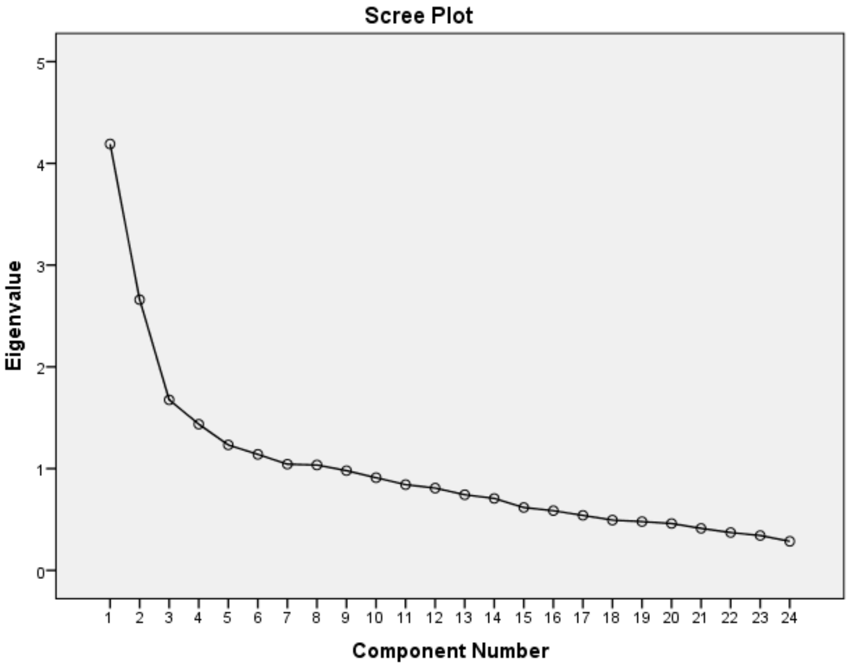

Supplement: Supplementary file 1 [file healthcare-14-01819-s001.zip › healthcare-4359894-supplementary.pdf]
